# Forgotten research from 19th century: science should not follow fashion

**DOI:** 10.1007/s10974-014-9399-4

**Published:** 2014-11-29

**Authors:** Stefan Galler

**Affiliations:** Department of Cell Biology, University of Salzburg, Hellbrunnerstr. 34, 5020 Salzburg, Austria

**Keywords:** Sliding filament theory, History of muscle research, Muscle birefringence, Cross-striated muscle

## Abstract

The fine structure of cross-striated muscle and its changes during contraction were known already in considerable detail in the 19th century. This knowledge was the result of studying birefringence properties of muscle fibres under the polarization microscope, a method mainly established by Brücke (Denk Kais Akad Wiss Math Naturwiss Cl 15:69–84, 1858) in Vienna, Austria. The knowledge was seemingly forgotten in the first half of the 20th century before it was rediscovered in 1954. This rediscovery was essential for the formulation of the sliding filament theory which represents the commonly accepted concept of muscle contraction (A.F. Huxley and Niedergerke, Nature 173:971–973, 1954; H.E. Huxley and Hanson, Nature 173:973–976, 1954). The loss of knowledge was the result of prevailing views within the scientific community which could be attributed to “fashion”: it was thought that the changes of cross-striations, which were observed under the microscope, were inconsequential for contraction since other types of movements like cell crawling and smooth muscle contraction were not associated with similar changes of the fine structure. The basis for this assumption was the view that all types of movements associated with life must be caused by the same mechanisms. Furthermore, it was assumed that the light microscopy was of little use, because the individual molecules that carry out life functions cannot be seen under the light microscope. This unfortunate episode of science history teaches us that the progress of science can severely be retarded by fashion.

## Introduction

The 43rd European Muscle Conference in Salzburg, Austria, provided me with a valuable opportunity to look back at Austria’s history of science. This offered me surprises on the history of the discovery of the mechanism of muscle contraction, from which we have a lesson to learn.

**Fig. 1:**
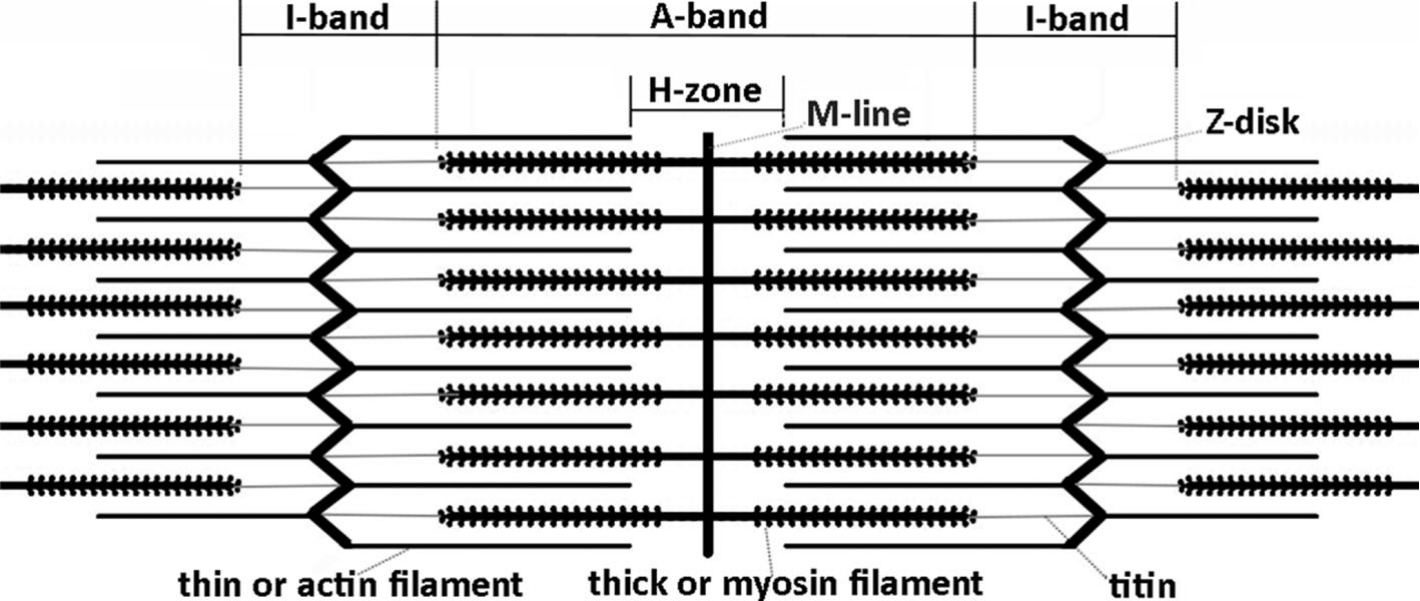
Schematic drawing for the fine structure of cross-striated muscle based on electron microscopy of H.E. Huxley (1957). The titin thread connecting the myosin filament with the Z-disk was found later (Maruyama et al. 1977) and named “connectin”. All the bands and lines labeled on top of the figure were first observed by Dobie (1849) using light microscopy. Brücke (1858) found that the I-band is isotropic whereas the A-band (and Z-disk) are anisotropic (or birefringent). Hensen (1869), Krause (1869), and Engelmann (1873a, b) enriched the knowledge about the Z-disk (*Zwischenscheibe* or Krause membrane), the Hensen-zone (H-zone) and the M-line (*Mittelscheibe)* especially by investigating the chemical persistence of these structures

Since the groundbreaking discovery of A.F. Huxley and Niedergerke (1954) and H.E. Huxley and Hanson (1954) 60 years ago, it has been generally accepted that muscle shortening occurs when the thin actin filaments in the isotropic I-band are drawn into the anisotropic A-band that consist of the thick myosin filaments (Fig. 1). This is called the “sliding filament theory”, and its conclusion was essentially based on microscopic observations demonstrating that the I-band width shortened linearly with a change in muscle length, whereas the A-band width remained the same. My findings on the history of the sliding filament theory were so overwhelming that I thought I should report on them here.

**Fig. 2:**
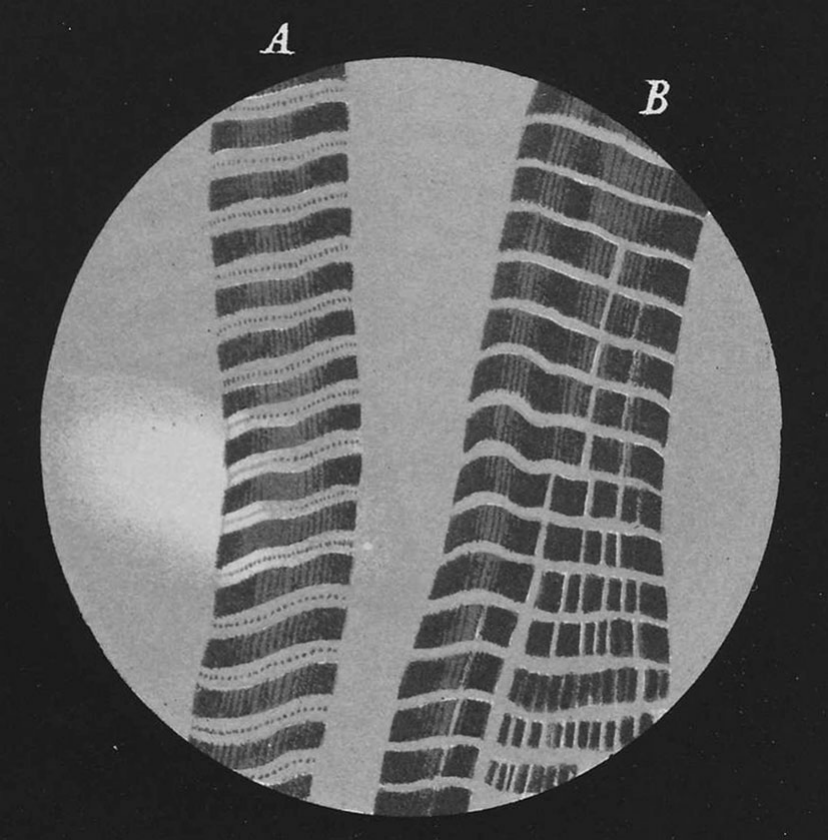
Polarization microscopic picture of muscle fibres originating from leg muscles of the water beetle *Hydrophilus piceus* (reproduction from the article of Brücke 1858). Further information is given in the text

At the beginning of my literature search, I came across the picture of two muscle fibres reproduced in Fig. 2. It shows cross-striated muscle fibres with a regular sequence of the dark A-band and the light I-band observed under a polarized-light microscope. In (A) the fibre seems to be more stretched than in (B). The width of the A-band is similar in (A) and (B), however, the width of the I-band is much narrower in (B) than in (A). Furthermore, the broad I-band in (A) shows a dark line at its center (Z-disk). Thus, this picture appears to be a good example demonstrating that muscle shortening is associated with a reduction in the width of the I-band, but not in the A-band.

Surprisingly, the picture shown in Fig. 2 was published in 1858. It came from *Denkschriften der kaiserlichen Akademie der Wissenschaften,* a book series which was published in Vienna, Austria, since 1850. The picture was included in the chapter *Untersuchungen über den Bau der Muskelfasern mit Hülfe des polarisierten Lichtes* (Investigations of the structure of muscle fibres with polarized light) in volume 15 (pp. 69–84) contributed by Ernst Brücke (1819–1892), a professor of physiology at The University of Vienna.

## Ernst Brücke

Ernst Brücke was a scientist of great merit and high reputation. He regularly communicated with other famous scientists of the 19th century and was knighted to *Ernst von Brücke* in 1873. Together with Carl Ludwig (Leipzig, 1816–1895), Hermann von Helmholtz (Berlin, 1821–1894) and Emil Du Bois-Reymond (Berlin, 1818–1896), Ernst Brücke was one of the foremost proponents for the theory that life was driven exclusively by physicochemical forces, and not by an additional vital force which is specific for living matter.

Brücke’s picture (Fig. 2) was a drawing reproduction of his observations with the light microscope. Microscopic photography was not yet established in the 19th century. How was it possible to observe such a detailed muscle structure as early as in 1858?

## Light microscopy in 19th century

In the middle of the 19th century, microscopes with exceptional quality were constructed in the workshop of Simon Plössl (1794–1868) in Vienna. Steiner and Schulz (2006) compared Plössl microscopes with modern ones and concluded that Plössl microscopes reached almost the modern standard for the sharpness and contrast at the central area of the picture. An important character of Plössl microscopes was their large focal depth, which allowed investigators to examine thick preparations like muscle fibres. Brücke used a Plössl microscope equipped with two Nicol prisms—one near the light source to polarize the light, and the other over the ocular for analyzing the rotation of the polarized light. With this equipment Brücke was able to study birefringence (anisotropy) of the muscle tissue. Brücke transformed birefringence signals into a colour image by using mica laminations of precise thickness as an object slide. This invention helped him distinguish between signals of transparency and birefringence, which was necessary to obtain clean-cut results of the muscle structure. Brücke’s investigations were helped by the use of muscle preparations with long sarcomeres. He mostly used preparations from leg muscles of the water beetle *Hydrophilus piceus*, which have sarcomere lengths ranging from 3.0 to 5.8 µm at rest (Edwards et al. 1954).

## Main findings of Ernst Brücke

When Brücke started his studies, some details of the fine structure and the birefringence property of skeletal muscle tissue were already known (e.g. Boeck 1839; Bowman 1840; Dobie 1849). In his 1858 article, Brücke described for the first time that only one of the two bands, namely the more refractive band (A-band) was birefringent. Most importantly, he found that the birefringence was uniaxial and parallel to the muscle fibre; it did not change when the fibre was activated, and it disappeared after treatment with acid or alkali. From these observations Brücke concluded that the A-band was composed of many birefringent rods lying in parallel with the fibre axis. He further concluded that during contraction the rods remained parallel; they did not change their shape but only their arrangements.

Brücke investigated mainly muscle preparations treated in ethanol, followed by turpentine oil, and stored in dammar resin varnish (Fig. 2). In addition, he studied fresh preparations taken from legs of *Hydrophilus piceus*. Such fresh preparations exhibit spontaneous contraction waves, which slow down with time, allowing easier observation under the microscope. Although the I-bands were very narrow in intact preparations, Brücke observed that the contraction waves caused the cross-striations to move closer together. Furthermore, he reported that the I-band widths were largest when shortening was not allowed before fixing the preparation.

## Follow-up studies

The observation, that the A-bands remained constant in length during contraction, whereas the I-bands shortened, was explicitly reported later by Krause (1869) for the first time. Engelmann (1873a, b) even found that not only the I-band width, but also the width of the brighter zone in the middle of the A-band, so called H-zone (Fig. 1), changed linearly with the fibre length. Furthermore, it was commonly assumed at that time that the force which causes contraction was generated in the A-band, whereas the I-band was a passive structure (Engelmann 1873b; Hermann 1879).

## Negative progress

I finally found an excellent summary of the microscopic investigations on muscle of the 19th century in the book *Reflections on Muscle* by A.F. Huxley (1980). Huxley mentioned that almost all of these fantastic works were completely forgotten in the first half of the 20th century. I would like to summarize the cause of this unwarranted loss of knowledge and some further circumstances which retarded the formulation of a sliding filament theory as follows.

1. In studies with insect abdominal muscles, contraction waves with extreme local shortenings were observed (Engelmann 1873b). These shortenings showed a considerable reduction of the A-band length.
2. The theories for the mechanism of muscle contraction which were proposed by Krause (1869) (electric attractions of A-bands), by Engelmann (1873b) (asymmetric swelling of the A-band) and others (Hermann 1879) were not satisfying in a way that they encouraged further investigations. The possibility that not only the A-band, but also the I-band was composed of filaments was never proposed in the 19th century, and therefore, the idea of the sliding filament theory never came across.
3. In the 1870s, the theory started to become prevalent that all types of movements associated with life must be based on the same mechanisms (e.g. Hermann 1879, 1886). Thus, it was thought that the elementary mechanism of contraction was the same for crawling movements of cells, for smooth muscle contraction, and for striated muscle contraction. As a consequence of this view, scientists came more and more to the conclusion that changes of fine structure of striated muscles were inconsequential for contraction because all other types of movements were not associated with similar structural changes visible under the microscope. Textbooks of physiology started to ignore the fine structure of the muscle. Thus, descriptions on muscle fine structure steadily decreased with time (Hermann 1886; Verworn 1915; Höber 1930).
4. At the beginning of the 20th century, the new generation of scientists seemingly believed that life functions were based on molecules which could not be seen under the microscope, and therefore, microscopy was of little use for explaining life functions. As a consequence, old microscopy studies on muscle fibres were neglected and microscopy was hardly used for further studies of muscle function. Moreover, the few studies carried out in the initial decades of the 20th century revealed misleading results. Hürthle (1909) reported for leg muscles of *Hydrophilus* that shortening was associated with the A-band, but not with the I-band. This was confirmed for frog muscle preparations by Holz (1932) and Buchthal et al. (1936). Because in these studies living muscle preparations were investigated by cine-photomicrography, their results appeared to have been more reliable than those of old studies of the 19th century which mainly used fixed preparations. However, as pointed out by A.F. Huxley (1980), the results of Hürthle (1909), Holz (1932) and Buchthal et al. (1936) deviated partly because of the very short initial muscle length (extreme contractions) and partly because of optical artefacts due to use of too thick muscle fibres in ordinary light microscopy. These studies were accepted by the scientific community at the time; consequently, the knowledge of the 19th century about changes in muscle fine structure during contraction completely disappeared.

One may argue that the loss of knowledge about muscle fine structure was not very momentous, because the concept of sliding filaments as formulated in 1954 would not have been possible earlier. Certainly, essential knowledge was missing: Most importantly, Straub (1942) showed that the contractile system is composed not by one but by two proteins, actin and myosin. Therefore, the muscle protein known as “myosin” since its first isolation by Kühne (1864) was actually the actomyosin complex. Furthermore, ATP was discovered in 1929 (Lohmann 1929; Fiske and SubbaRow 1929) and its role for muscle contraction was elucidated. Engelhardt and Ljubimowa (1939) discovered that actomyosin cleaves ATP enzymatically. Furthermore, Szent-Györgyi (1941–1942a, b) found that ATP reduces the viscosity of actomyosin and induces actomyosin threads to contract. Finally, H.E. Huxley (1953) was able to localize the filaments formed by myosin and actin within a sarcomere as shown in Fig. 1 with electron microscopy.

While it is true that the concept of sliding filaments would not have been possible without this new knowledge, it is worth asking how science could have progressed if the light microscopy work of the 19th century had not been forgotten. This question is all the more justified, since important biochemical results of the 19th century passed unheeded as well: As reviewed by Perry (2003), Straub’s discovery of actin in 1942 was actually already anticipated in part by Halliburton (1887). Thus, science already at that time was not far from the conclusion that Kühne’s “myosin” was composed of two proteins associated with the A- and I-band.

## Science should not follow fashion

The above report demonstrates that the negative developments in the forefront of the sliding filament theory were not only due to unfortunate experimental conditions, but they were essentially due to prevailing views within the scientific community which could be attributed to “fashion”. Therefore, this unfortunate episode of science history teaches us that the progress of science can severely be retarded by fashion.
